# Near infrared readouts offer sensitive and rapid assessments of intestinal permeability and disease severity in inflammatory bowel disease models

**DOI:** 10.1038/s41598-020-61756-y

**Published:** 2020-03-13

**Authors:** Liang Zhang, Craig D. Wallace, Jamie E. Erickson, Christine M. Nelson, Stephanie M. Gaudette, Calvin S. Pohl, Samuel D. Karsen, Gricelda H. Simler, Ruoqi Peng, Christopher A. Stedman, F. Stephen Laroux, Marc A. Wurbel, Rajesh V. Kamath, Bradford L. McRae, Annette J. Schwartz Sterman, Soumya Mitra

**Affiliations:** 1 AbbVie Bioresearch Center, Worcester, MA 01605 USA; 2 AbbVie Inc, North Chicago, IL 60064 USA

**Keywords:** Gastroenterology, Imaging

## Abstract

Intestinal permeability and neutrophil activity are closely linked to inflammatory bowel disease (IBD) pathophysiology. Here we discuss two techniques for assessing permeability and neutrophil activity in mouse IBD models using near infrared (NIR) detection. To address the limitation of visible light readouts—namely high background—IRDye 800CW was used to enable rapid, non-terminal measurements of intestinal permeability. The increased sensitivity of NIR readouts for colon permeability is shown using dextran sulfate sodium (DSS) and anti-CD40 murine colitis models in response to interleukin-22 immunoglobulin Fc (IL22Fc) fusion protein and anti-p40 monoclonal antibody treatments, respectively. In addition to enhanced permeability, elevated levels of neutrophil elastase (NE) have been reported in inflamed colonic mucosal tissue. Activatable NIR fluorescent probes have been extensively used for disease activity evaluation in oncologic animal models, and we demonstrate their translatability using a NE-activatable reagent to evaluate inflammation in DSS mice. Confocal laser endomicroscopy (CLE) and tissue imaging allow visualization of spatial NE activity throughout diseased colon as well as changes in disease severity from IL22Fc treatment. Our findings with the 800CW dye and the NE probe highlight the ease of their implementation in preclinical IBD research.

## Introduction

Ulcerative colitis (UC) and Crohn’s disease (CD) are two main forms of both acute and chronic inflammatory bowel diseases (IBD) with complex disease etiology. While multiple anti-inflammatory treatment strategies are available to patients, there is active interest in the development of therapeutic strategies that improve epithelial repair and barrier function to reduce pro-inflammatory burdens^1–3^. Several clinical studies suggest alterations in paracellular permeability and tight junction functions are key events in the pathogenesis of IBD^3–5^. In addition to traditional readouts of disease severity such as body weight, colon length, and pathology score, screening and assessment of intestinal tight junction permeability is an increasingly important measurement in animal colitis models. Ratiometric measurements of sugar concentrations in urine provide gastrointestinal permeability data in the clinic, but data are highly variable and require significant urine collection at multiple timepoints^6,7^. Other strategies utilize small molecule such as radiolabeled EDTA but require ionizing radiation and are more costly^8^. Due to ease of handling, *in vivo* assessment of intestinal epithelial permeability in pre-clinical models largely relies on measuring serum fluorescein isothiocyanate (FITC)-labeled dextrans that are orally gavaged. However, 4 kDa FITC-dextran detection has limited sensitivity due to high blood autofluorescence and may prevent accurate differentiation between treatment groups in dose-response experiments, where absolute differences in fluorescence intensity may be low and/or near the limit of detection. Low molar absorptivity in addition to high tissue autofluorescence also mandate high doses of FITC-dextran for detection^9–11^ (often >500 mg/kg in mice), precluding its use in the clinic. Owing to low tissue autofluorescence, deep tissue penetration depth, and high spatial resolution, near infrared (NIR) and far-red fluorophores have been used for disease screening by elucidating both tissue and cellular level distribution of administered therapeutics^12–16^. Here, we explore the utility of a commercially available and clinically-adopted NIR dye, IRDye 800CW (herein referred to as 800CW), to assess intestinal permeability in preclinical models of IBD^17^.

In addition to enhanced permeability, neutrophil recruitment and subsequent activation are among the earliest inflammatory responses in the mucosa to limit microorganism invasion^18^. Upon activation, neutrophils release neutrophil elastase (NE), an enzyme associated with disrupting epithelial barrier function through E-cadherins and zonula occludens-1 degradation. Consequent mucosal dysfunction is thought to play a role in pathogenesis of IBD^18,19^. Endogenous protease inhibitors regulating NE become inactivated at sites of inflammation and as a result, elevated levels of NE activity has been measured both systemically and locally in colonic mucosal tissue in both rodents and humans^20^. Systemic NE inhibitors have hindered disease progression in rodent colitis models and there is active research pursuing NE inhibitors as anti-inflammatory therapies with ongoing clinical trials in lung and cardiovascular diseases^21^. Several fluorogenic probes are available for imaging NE and corroborating imaging data with translational measurements of disease severity can greatly aid in the design of novel treatments. These imaging agents comprise of a peptide substrate linking two intramolecularly quenched NIR fluorophores. Fluorescence intensity is recovered upon protease cleavage of the substrate. Based on its performance to specifically elucidate NE activity in tumors and acute lung injury models as well as robust *in vitro* characterization^22,23^, NE680 FAST (herein referred to as NE680) was selected to image spatial NE activity in colitis models. The ability to visualize and quantify NE *in vivo* and *ex vivo* provides a promising path for monitoring colonic inflammation and disease severity.

Preclinical small animal models of IBD focus on active induction, adoptive transfer, genetic modification, etc. The dextran sulfate sodium (DSS) colitis model is a well-established animal model of mucosal inflammation that has been used extensively in IBD preclinical studies and presents features that closely resemble human UC^9,24–26^. Chemical damage to the colonic epithelial layer allows bacteria and other proinflammatory contents to reach underlying tissue. Using this model, we evaluate 800CW permeability and NE680 disease activity imaging in response to treatment with an IL22Fc fusion protein promoting epithelial repair^27^. To our knowledge, this is the first use of a NE sensitive marker to detect and quantify inflammation in a rodent colitis model. Originally described by Uhlig *et al*., the anti-CD40 agonist colitis model is a useful acute model established in immune-compromised mice for interrogating myeloid-driven gut inflammation^28,29^. More recently, Wurbel *et al*. demonstrated a dose-dependent model of anti-CD40 colitis in wild-type immunocompetent mice^30^. In this acute anti-CD40 model, where inflammation but no epithelial erosion is observed via histopathology, FITC-dextran is unable to measure changes in colon permeability. We hypothesize that these small changes in permeability accompanying the acute response will be detectable via 800CW, and selected this model to demonstrate the increased sensitivity of NIR detection.

## Results

**Figure 1:**
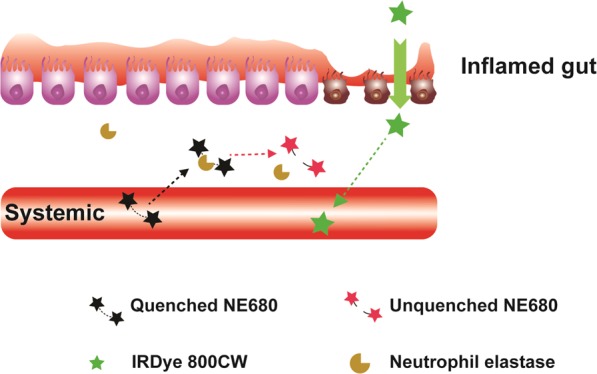
Schematic depicting 800CW and NE680 transport in inflamed gut. Intravenously dosed Neutrophil Elastase 680FAST is optically silent until protease cleavage at the site of disease, allowing fluorescence to recover. Paracellular transport of orally dosed 800CW across tight junctions in inflamed colonic tissue allows for systemic measurements.

**Figure 2:**
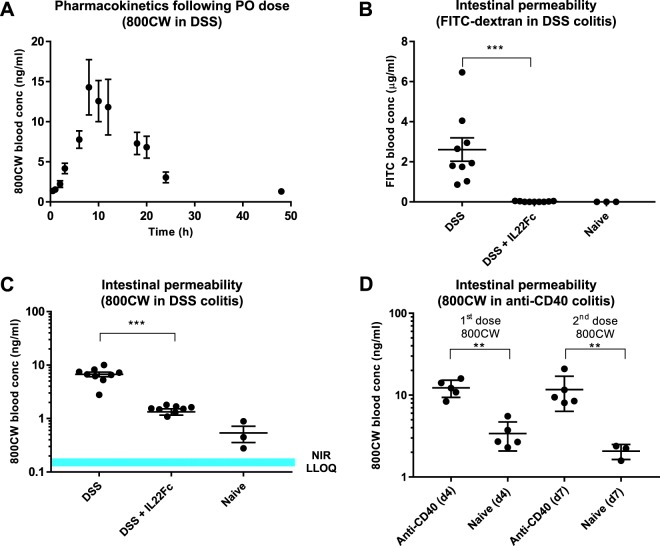
Characterization and validation of 800CW as a permeability readout in mouse models. (**A**) Pharmacokinetics of orally dosed 800CW carboxylate in mouse DSS. For each timepoint, n = 10. (**B**) FITC dextran permeability measurements in murine DSS colitis. Naive animal measurements are below the limit of detection. For DSS, n = 9; for DSS + IL22Fc, n = 10; for naive, n = 3. (**C**) 800CW permeability measurements demonstrating detectable signal in all groups. For DSS, n = 9; for DSS + IL22Fc, n = 10; for naive, n = 3. (**D**) Repeat dose and blood measurements of 800CW in an anti-CD40 mouse model of colitis demonstrating increased gut permeability in diseased animals. For anti-CD40, n = 5; for naive, n = 3. All values presented are the mean ± S.E.M. One-way ANOVA was used to analyze variation; ***P < 0.001, **P < 0.05.

The optical properties of NIR and far red fluorophores allow for improved sensitivity for colon permeability and NE activity imaging. 800CW carboxylate and fluorogenic NE680 were orally and intravenously administered, respectively. 800CW is absorbed from the colon to blood, where it can be quantified via blood sampling. NE680 is administered intravenously; following distribution into the target disease tissue, NE cleaves the enzyme-sensitive peptide linkage, unquenching the VT680 fluorophore (Fig. 1). To validate 800CW as a colon permeability marker, C57BL/6 mice treated with DSS in drinking water were co-dosed with both FITC-dextran and 800CW orally at 600 and 2 mg/kg, respectively (Fig. 2A,B,C). We hypothesized that a successful NIR permeability marker will be stable in the stomach, have adequate colon bioavailability for detection, exhibit blood stability, and clear rapidly through renal filtration. Sample collection 8 h post dosing indicated higher blood concentrations of FITC dextran and 800CW in DSS treated vs naive control animals (FITC: 2.6 μg/ml vs below detection limits in naive controls, *P* < 0.001; 800CW: 6.7 ng/ml vs 0.5 ng/ml, *P* < 0.001). As IL22 signaling in the intestine has been shown to promote mucosal wound healing^31^, animals treated with DSS were also prophylactically dosed with IL22Fc to quantify any concomitant impact on permeability. This prophylactic treatment reduced dextran permeability by >95% reduction compared to 85% reduction for 800CW (FITC: 0.02 μg/ml, near detection limits; 800CW: 1.3 ng/ml). Both FITC-dextran and 800CW have radii greater than 4 Å (hydrodynamic radius 12 Å and 7 Å for FITC-dextran and 800CW, respectively, using polyethylene glycol (PEG) oligomer approximations^32^). Lumen to blood transport is likely restricted to leak pathways for this molecular size^33^, suggesting differences in permeability reduction in treatment groups are due to FITC nearing the limit of detection. In addition to matching the qualitative trends and relative decreases in permeability with IL22Fc treatment, 800CW absorption is quantifiable in naive controls, a distinction that FITC-dextran cannot distinguish due to detection limits. Serial sampling of whole blood allowed for full pharmacokinetic characterization of orally dosed 800CW in DSS colitis models (Fig. 2A). While whole blood concentrations from naive animals remained constant during the time course (2 ng/ml), systemic C_max_ for orally dosed 800CW occurs at approximately 8 h (13 ng/ml) for DSS treated animals and agrees well with previously measured transit times in mouse colon^34^. Pharmacokinetic data identified a sampling window and suggested possible repeat oral dosing after 48 h to enable longitudinal measurements in the same animal. This was shown using an anti-CD40 agonist acute colitis model^30^, where body weight data from day 4 through day 7 allow for multiple doses of 800CW (Supplementary Fig. S5). Animals received 120 μg doses of 800CW on days 4 and 7 of disease (Fig. 2D). Anti-CD40 and naive animals displayed statistically significantly different colonic permeability (12.3 ng/ml vs 3.4 ng/ml for day 4, *P* = 0.004; 11.7 ng/ml vs 2.1 ng/ml for day 7, *P* = 0.007). No statistically significant differences in permeability were observed between day 4 vs day 7.

**Figure 3:**
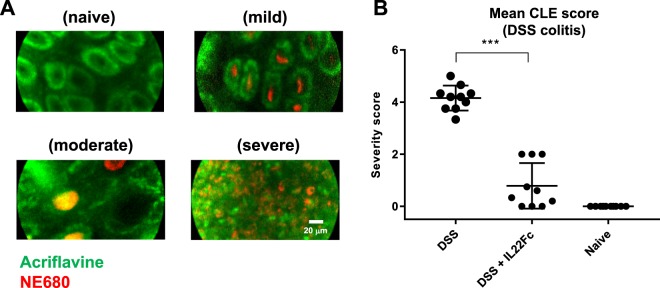
Neutrophil Elastase 680 and acriflavine CLE with severity scoring. (**A**) Representative CLE video frames for different disease severity features. Acriflavine signal in green; NE680 signal in red. (**B**) Scores determined by video analysis with mean scores plotted. For DSS, n = 10; for DSS + IL22Fc, n = 10; for naive, n = 10. Values presented are the mean ± S.D. One-way ANOVA was used to analyze variation; ***P < 0.001.

**Figure 4:**
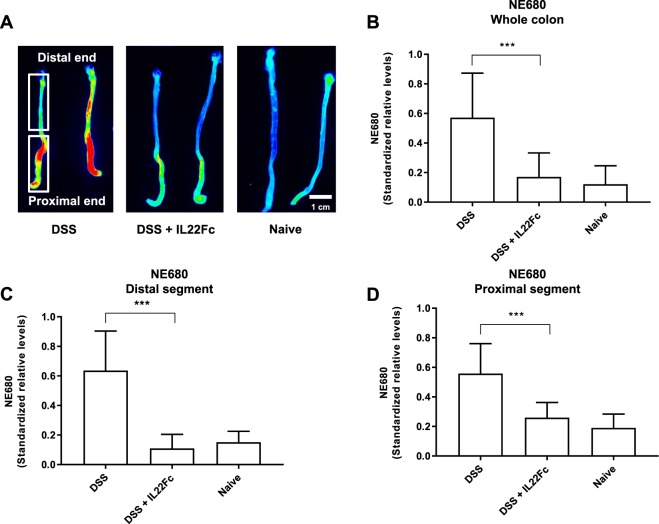
Neutrophil Elastase 680 *ex vivo* colon imaging. (**A**) Resected mouse colon highlighting different levels of neutrophil activity by treatment group. (**B**) Whole organ ROI analysis show decrease in NE680 signal for DSS + IL22Fc treated animals. (**C,D**) Segmented analysis of distal and proximal colon. For DSS, n = 20; for DSS + IL22Fc, n = 20; for naive, n = 20. All values presented are the mean ± S.D. One-way ANOVA was used to analyze variation; ***P < 0.001.

Neutrophil elastase and macrophage activity in mouse DSS colitis in response to IL22Fc treatment highlights the relationship between intestinal permeability and changes in inflammation. NE680 and acriflavine imaging via confocal laser endomicroscopy (CLE) revealed characteristic disease features (Fig. 3A). CLE video frames up to the distal 2 cm of colon were captured, analyzed, and assigned a severity score (Table 1) for animals treated with DSS only, DSS + IL22Fc, and no treatment (Fig. 3B). The colons were resected and imaged macroscopically *ex vivo* to corroborate the *in vivo* CLE findings (Fig. 4A). On the organ level, NE680 intensities were higher in the DSS only group and reduced markedly for DSS + IL22Fc animals (Fig. 4B; *P* < 0.001). NE680 image analysis also suggested no statistical differences between DSS + IL22Fc and naive controls (Fig. 4B). Signal in the proximal colon is consistently higher than the distal colon within the same animal; this is likely due to increased tissue surface area in the proximal colon (Fig. 4A). Based on these observations, segmented image analysis of NE680 in the distal and proximal colon regions was performed. However, no statistical differences were observed between DSS + IL22Fc vs naive groups in either the distal or proximal colon segments (Fig. 4C,D). The *ex vivo* NE680 results highlight the challenges of directly using pixel intensity-based analyses on the macroscopic organ level, where tissue thickness and vascularization may differ between regions of interest within the same organ.

**Table 1:** Confocal Laser Endomicroscopy (CLE) Scoring Criteria.

Description	Score
No change in crypt architecture and absence of NE680 signal	0
Healthy crypt structure; NE680 signal in crypt lumen	1
>50% healthy crypt structure; high NE680 signal in crypt lumen; presence of collapsed crypts	2
Significant presence of collapsed crypts; high NE680 positive area	3
>50% of crypts lack acriflavine staining; high NE680 signal in crypt lumen; presence of individual NE680 positive cells within erosions	4
Only NE680 positive cells within erosions; significant crypt loss	5

**Figure 5:**
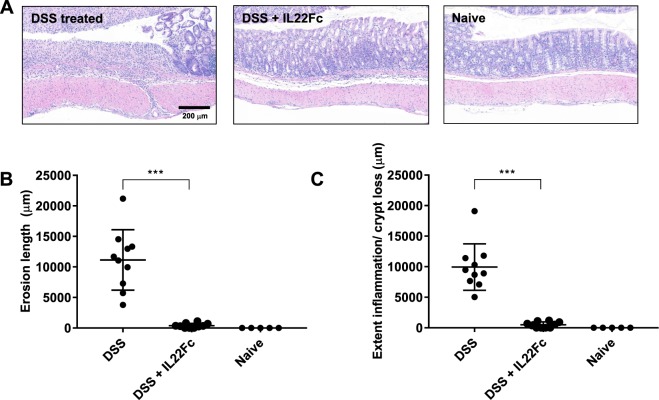
Histological assessment of epithelial erosions in C57BL/6 mouse colon. (**A**) Characteristic H&E stains: left: ulceration of the epithelial layer, crypt damage, and infiltration of inflammatory cells into the colon mucosa in DSS treated animals; middle: reduction in epithelial damage for DSS + IL22Fc treated animals; right: naive animal. (**B,C**) Erosion length, extent of inflammation, and crypt loss analyses suggest DSS + IL22Fc treatment provides epithelial protection at near naive levels. For DSS, n = 10; for DSS + IL22Fc, n = 10; for naive, n = 5. All values presented are the mean ± S.D. One-way ANOVA was used to analyze variation; ***P < 0.0001.

**Figure 6:**
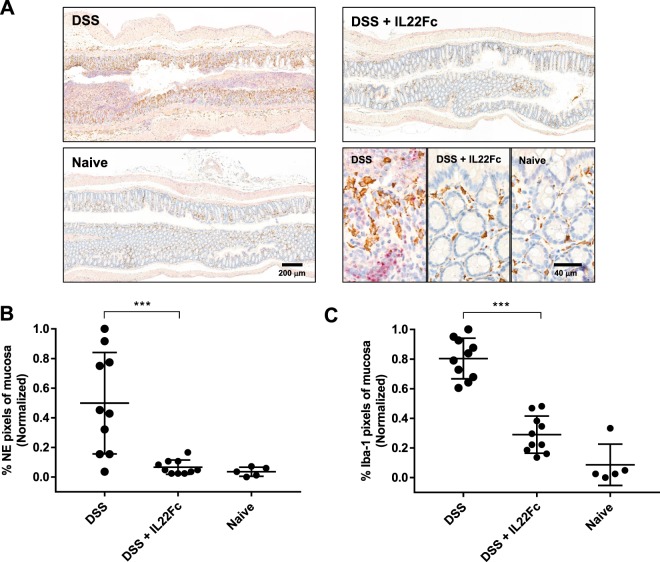
Neutrophil elastase and pan macrophage immunohistochemistry in DSS colon. (**A**) Anti-neutrophil elastase (red) and Iba-1 staining (brown) for DSS treated, DSS + IL22Fc treated, and naïve animals. (**B,C**) Anti-NE and Iba-1 image analyses show elevated macrophage infiltration and NE staining in DSS treated animals and a reduction with DSS + IL22Fc treatment. For DSS, n = 10; for DSS + IL22Fc, n = 10; for naive, n = 5. All values presented are the mean ± S.D. One-way ANOVA was used to analyze variation; ***P < 0.001.

Traditional histological measurements and immunohistochemistry (IHC) helped corroborate the imaging results. The CLE scoring system (Table 1), which combines crypt architecture integrity with NE activity, was compared to histological measurements of colonic epithelial erosion damage (Fig. 5A). Representative hematoxylin and eosin (H&E) stained colon sections and associated regions of epithelial erosions in DSS treated and DSS + IL22Fc treated groups agree well with CLE findings (Fig. 5B,C). In DSS groups, a micrograph of junction of a mucosal ulcer (Fig. 5A, left) indicates complete loss of surface epithelium and crypt architecture. Moderate infiltrates comprised of neutrophils, eosinophils and macrophages extend to the submucosa and dissect through muscularis and perivascular space. A representative micrograph for DSS + IL22Fc animals (Fig. 5A, center) shows intact mucosal epithelium with mild acute inflammatory infiltrates in the lamina propria and submucosa. Goblet cells were maintained at naive levels in the DSS + IL22Fc group. However, 50% of the same cohort received a mild score (1–2) via CLE due to presence of NE680 positive signal in the crypts. We further evaluated NE and macrophage presence using whole slide image analysis of colon tissue sections immunolabeled with an anti-NE antibody (Fig. 6A). Anti-NE image analysis highlighted differences between DSS only vs DSS + IL22Fc treated groups (*P* < 0.001) but not between DSS + IL22Fc vs naive animals (Fig. 6B) and agreed well with fluorescence imaging data. Area-based image analysis using ionized calcium binding adaptor molecule-1 (Iba-1) has been used in rodent models of inflammation as a macrophage marker and Jin *et al*. showed agreement between flow cytometry using CD45^high^/CD11b + microglia/macrophage staining and Iba-1 IHC image analysis^35,36^. Thus, the increase of monocytes and macrophages accompanying intestinal permeability changes was investigated in mouse DSS via Iba-1 IHC (Fig. 6A). The analysis revealed elevated macrophage infiltration in the colon mucosa of DSS treated vs DSS + IL22Fc treated animals (*P* < 0.001) as well as DSS + IL22Fc vs naive groups (*P* < 0.001, Fig. 6C). These fold-changes agree well with changes in mRNA levels of CD64 and Arginase 1 in DSS colitis^37^.

**Figure 7:**
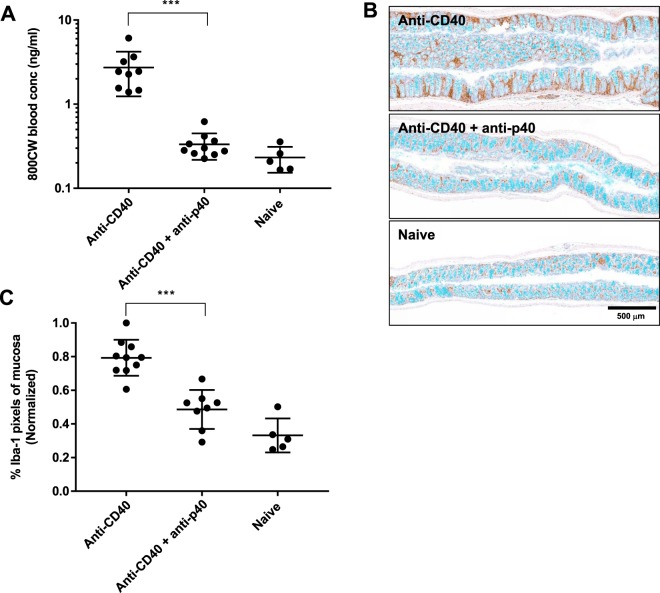
Mucosal inflammation and gut permeability in CD40 colitis. (**A**) Elevated blood levels of orally dosed 800CW were observed in anti-CD40 treated animals. A reduction in permeability and blood levels was observed in anti-CD40 + anti-p40 treated animals and were similar to naive animals. For anti-CD40, n = 9; for anti-CD40 + anti-p40, n = 10; for naive, n = 5. (**B**) Visible signs of erosions for analysis were not observed in anti-CD40 treated animals. Rather, Iba-1 IHC was performed to quantify macrophage infiltration. Iba-1 IHC in brown; mucin staining in light blue. (**C**) Iba-1 image analysis suggests statistically significant higher staining in anti-CD40 animals vs anti-CD40 + anti-p40 animals. For anti-CD40, n = 10; for anti-CD40 + anti-p40, n = 8; for naive, n = 5. All values presented are the mean ± S.D. One-way ANOVA was used to analyze variation; ***P < 0.001.

Based on 800CW permeability in the anti-CD40 colitis model (Fig. 2D), where there is inflammation but minimal epithelial erosion/disruption, we further explored the relationship between mucosal inflammation and gut permeability^28^. To our knowledge, gut permeability in this model has not previously been investigated, possibly due to lack of sensitivity using FITC-dextran. Anti-CD40 treated animals showed elevated colonic permeability vs naive animals (2.7 ng/ml vs 0.3 ng/ml, Fig. 7A). Anti-CD40 + anti-p40 treatment reduced permeability>13-fold compared to the anti-CD40 group (*P* < 0.001). Although relative decreases in permeability may be more useful than absolute decreases for assessing disease severity and/or treatment progression, lower systemic 800CW was observed in naive SCID controls (0.2 ng/ml) compared to naive C57BL/6 (1.4 ng/ml) from DSS studies; this is likely due differences in blood sampling times between the colitis models. Decreased systemic 800CW for the anti-CD40 + anti-p40 treated group is consistent with the anti-inflammatory response accompanying anti-p40 treatment as supported by Iba-1 image analysis (Fig. 7B,C).

To assess the scalability of a NIR permeability readout, three C. macaques were orally dosed with 800CW dye (Supplementary Fig. S11). Intestinal transit times are significantly longer in non-human primates (NHP) compared to rodents, and systemic pharmacokinetics for sick primates lacked the bimodal absorption profile observed in sick mice (Fig. S11). This highlights probe design challenges due to species-specific oral absorption kinetics.

## Discussion

Many preclinical and clinical readouts of disease severity and disease diagnosis for IBD employ dye-based detection. Some commonly used techniques include FITC-dextran for colonic permeability^9,38^, cresyl violet and acriflavine in CLE for superficial cell feature staining^39^, and contrast agents such as methylene blue in chromoendoscopy for neoplasia detection^40^. Limitations of detection systems can make visible light readouts challenging to interpret, and white light endoscopic systems may not simultaneously assess tissue morphology and disease activity. Near infrared detection is a popular optical imaging approach due to low autofluorescence in biological tissue. There are numerous commercially available NIR fluorophores with a variety of chemical functionalization for covalent conjugation, hence an increased use of these fluorophores in the clinic for screening disease tissue and in preclinical research for biodistribution and pharmacokinetics^41–43^.

Paracellular transport across tight junctions is tightly regulated to limit unwanted microbiota from internal compartments^44^. As a result, tight junction permeability is selective for molecules with strict size and charge requirements^33,45^. For example, *in vitro* flux of different sized PEG molecules via pore pathways (ion selective) revealed markedly decreased transport for sizes greater than 4 Å. Practically, these limitations require high oral doses of FITC-dextran (frequently > 400 mg/kg) and/or significant sample collection for reliable and reproducible detection. Desired traits of a permeability marker include stomach and blood stability, adequate colon bioavailability, and rapid renal clearance. With these properties in mind, an analogous 4 kDa 800CW-dextran was initially investigated as a FITC-dextran replacement (Supplementary Fig. S2). FITC-dextran and 800CW-dextran showed similar trends in co-dosed experiments, but the absolute signal for 800CW-dextran in DSS treated animals was lower than predicted assuming a similar bioavailability as FITC-dextran. The absorption spectrum for 800CW-dextran revealed a blue-shifted shoulder at 700 nm, suggesting intramolecular quenching and reduced fluorescence due to high fluorophore degree of label (data not shown). To alleviate these issues, the inert, polyanionic 800CW carboxylate dye was used instead. Several other orally dosed hydrophilic, polyanionic molecules have shown measurable blood concentrations including heparins and NIR molecular targeting imaging agents^12,46–48^. Although 800CW (1 kDa) has 4-fold lower molecular weight (MW) compared to the dextran conjugates, it likely avoids ion selective pathways for oral absorption, unlike carboxyfluorescein (~4–5 Å). The decrease in molecular weight (1 kDa for 800CW vs ~4 kDa for 800CW-dextran) minimally impacted an already low bioavailability, removed polydispersity, reduced the oral dose 100 to 300-fold, and improved signal while reducing cost and time due to synthesis.

Neutrophil infiltration is a prominent histological feature of IBD^49^. Ginzberg *et al*. demonstrated in a colonic epithelial cell line that neutrophil-derived proteases, such as NE, mediate the disruption of epithelial apical junctions during transmigration^19^. Several findings have also reported on the relationship between IBD and NE^50^, and fecal NE levels have been shown to correlate with disease activity in IBD patients^51,52^. Thus, accurately measuring NE and disease activity remains a priority for assessing acute intestinal inflammation in the clinic. Currently, biomarker-based readouts such as Vitamin D, blood immune cell ratios, and fecal neutrophil-derived proteins are commonly used^53–56^. These data correlate well with total disease activity but do not provide disease spatial distribution. Imaging-based measurements of immune cell activity, on the other hand, have the potential to identify disease heterogeneity and visualize therapeutic efficacy. To address this, we use a combination of acriflavine and NE680 to simultaneously capture crypt architecture and macroscopic NE activity *in vivo* using CLE. In the preclinical approach, whole organ resection following CLE enables tissue and cellular level analyses. The CLE findings and *ex vivo* imaging revealed marked increases in colonic NE activity in DSS-treated mice followed by a reduction in disease activity upon IL22Fc treatment. NE680 imaging also agreed well with histological analysis of epithelial erosions. Interestingly, the imaging data suggest decreased inflammation downstream of IL22Fc treatment. IL22 is reported to play a key role in maintaining mucosal barrier function, and while epithelial regeneration does not equate to a reduction in mucosal inflammation, the anti-inflammatory effects we observe with NE and Iba-1 analyses parallel the histological evidence of epithelial protection. Due to the lack of IL22 receptor expression on hematopoietic cells^57,58^, we hypothesize mucosal barrier recovery in response to IL22Fc treatment is responsible for the observed reductions in colonic inflammation and permeability. This is consistent with the ameliorative properties of IL22 on local intestinal inflammation in mouse colitis models^59,60^.

Applications of “smart” fluorogenic probes with enzyme sensitive linkages are increasingly popular for preclinical IBD research, where they have potential to be valuable screening and diagnostic tools^61,62^. Finnberg *et al*. used a NIR “smart” cathepsin probe in mouse DSS colitis to identify regions of increased inflammation and epithelial dysfunction^61^. Ding *et al*. observed strong agreement between probe-associated colon signal and histologic score of acute inflammation using cathepsin and matrix metalloproteinase (MMP) probes^62^. Along with our own findings in this work using CLE, fluorogenic probes hold potential as imaging biomarkers of colonic inflammation for clinical translation.

Anti-neutrophil elastase and Iba-1 IHC helped corroborate NE imaging data with myeloid regulation of gut permeability. While trends between DSS only and DSS + IL22Fc treated groups using either marker were similar, Iba-1 identified differences between DSS + IL22Fc animals vs naive animals. This agrees with the 2-fold change in 800CW permeability (Fig. 2C). To further understand the interplay between macrophage associated inflammation and permeability, we evaluated the impact of anti-p40 treatment in anti-CD40 colitis animals. The anti-CD40 model exhibits minimal epithelial damage and the mucosal inflammation is primarily driven by activated macrophages^28^. In this model, barrier disruption is secondary to myeloid activation in the initiation of colitis. Several clinical and pre-clinical studies have shown treatment with anti-p40 induces an anti-inflammatory response due to inhibition of both IL12 and IL23 cytokines that share the p40 subset^28,63,64^. Consistent with these reports, and as a validation of our hypothesis, we observe reductions in colon Iba-1 and intestinal permeability for the anti-CD40 + anti-p40 treatment group. Importantly, these small changes in permeability were undetectable using FITC-dextran (data not shown). The findings suggest that anti-p40 improves the epithelial barrier function with concomitant reductions in mucosal inflammation.

Several challenges must be addressed to scale NIR permeability probes from animal models to the clinic. For example, the data suggest animal to animal variability may be disease dependent, and the blood readouts have a higher variance in DSS vs anti-CD40 colitis. In the DSS colitis model, histopathology reveals heterogeneous distributions of multiple lesions in both proximal and distal colon several millimeters in size. Blood flow into the lumen from these lesions may interfere with passive diffusion of orally dosed permeability marker. Leakage of intravenously dosed fluorescein into crypt lumen is well-documented^39,65^ and normalizing permeability readouts to these convective elements may reduce readout variability. Second, we hypothesized a successful permeability marker is minimally absorbed in the small intestine relative to colon via a leak pathway (not ion selective) in order to maximize disease-specific signal. Non-human primate oral absorption kinetics revealed significantly higher 800CW oral bioavailability compared to naive mice (Fig. S11). The molecular structure of 800CW suggest a more dominant role for leak pathways in NHP compared to mice^33^. Improvements in probe design, such as increasing molecular radii to avoid leak pathway absorption in healthy primates, may better discern permeability differences for primate colitis models with an end goal towards clinical translation. Lastly, a clinically relevant permeability marker must exhibit no toxicity. In rats, no adverse effects were observed following 20 mg/kg administration^66^. The reactive form of the fluorophore has been used in protein conjugations for image-guided surgery to evaluate the feasibility of clinical translation^17,67,68^. The difference in 800CW dose required for fluorescent imaging and blood readouts must also be considered.

In conclusion, we developed novel readouts of intestinal permeability and NE activity using far-red and NIR probes in common preclinical colitis models. Favorable optical properties in this detection range enabled increased sensitivity and dynamic range for permeability measurements as well as reducing animal usage due to small samples needed for analysis. Confocal laser endomicroscopy with NE680 and acriflavine enabled *in vivo* disease severity scoring criteria that agree well with endoscopy, histopathology, and immunohistochemistry. NE680 imaging also provided spatial NE activity that agreed well with IHC image analysis. Although further progress in probe design is needed for human use, each probe offers important and independent assessments of disease status and presents potential translational strategies for aiding IBD research.

## Methods

### Materials

Dextran sulfate sodium was obtained from MP Biomedical (Santa Ana, CA). Fluorogenic probe NE680 FAST was obtained from Perkin Elmer (Waltham, MA). FITC-dextran 4 kDa and acriflavine were obtained from Sigma Aldrich (Milwaukee, WI). IRDye 800CW carboxylate and IRDye 800CW NHS were obtained from Li-Cor (Lincoln, NE). Amino dextran 3.4 kDa was obtained from FinaBio (Rockville, MD). Iba-1 Rabbit Polyclonal and neutrophil elastase rabbit polyclonal antibodies were obtained from Wako Chemicals (Japan) and Abcam (Cambridge, MA), respectively. Anti-mouse CD40 monoclonal antibody (Clone FGK4.5) was obtained from BioXCell (West Lebanon, NH). Mouse IL22Fc is a fusion protein comprised of murine interleukin-22 (IL22) linked to the Fc region of mutant mouse IgG1 via flexible serine glycine linker. Mouse IL22Fc was produced in human embryonic kidney cells (AbbVie, Worcester, MA). Anti-IL12/23p40 monoclonal antibody was produced in a hybridoma cell line obtained from ATCC (CRL-2358) by Harlan Bioproducts for Science, Inc. (Indianapolis, IN). Leica Bond Polymer Refine Detection kits for HRP and AP were obtained from Leica Biosystems (Newcastle, UK).

### Animals

Mice utilized in these studies were conducted at AbbVie to the standards of the Association for the Assessment and Accreditation of Laboratory Animal Care (AAALAC) standards. All studies with mice were performed according to approved protocols by AbbVie’s Institutional Animal Care and Use Committee (IACUC). Wild-type C57BL/6 and immunocompromised SCID mice were used for DSS and anti-CD40 colitis models, respectively. Briefly, seven-day acute murine colitis was induced either with 3% DSS in the drinking water in C57BL/6 mice or with a single IP injection of 5 mg/kg CD40 agonist antibody in SCID mice. Colitis in the SCID model is CD40 dose dependent and Wurbel *et al*. report intestinal inflammation and Iba-1 positive cells in the mucosa from day 3 to day 7^30^. Body masses were monitored, and animals were euthanized if >20% of the initial weight was lost. IL22Fc (2.5 mg/kg) was dosed intravenously on day 0 and day 3 of DSS treatment via the tail vein. Prophylactic treatment with 25 mg/kg of anti-IL12/23p40 was performed in anti-CD40 colitis mice using IP injections on day -1 and day 2 of disease.

Primates utilized in this study were female cynomolgus macaques of Chinese origin and were pair housed at AbbVie to AAALAC standards. All studies with primates were performed according to approve protocols by AbbVie’s IACUC (Protocol ID: 1705C0002).

### Permeability

To validate 800CW as a colon permeability marker in DSS colitis models, mice were fasted for 4 h, followed by a co-dose via oral gavage with 2 mg/kg 800CW and 600 mg/kg FITC-dextran. 600 mg/kg FITC dextran dosage determined by instrument detection limits and according to previously published doses. Animals were euthanized 8 h later and serum was collected for analysis following FITC-dextran protocols. FITC fluorescence (excitation/emission 495/519 nm) was quantified using a Biotek Synergy2 (Winooski, VT) and 800CW fluorescence (excitation/emission 774/789 nm) was measured using a Li-Cor Odyssey CLx (Lincoln, NE). Fluorescence was converted to serum concentration using a standard curve and relative changes in blood concentration were compared to validate 800CW permeability measurements. Post validation, fasting was removed and terminal serum was no longer collected for 800CW permeability. Instead, 10 μL whole blood was collected via tail nick and mixed with 20 μL of 10 mM EDTA in PBS. Blood samples were frozen immediately at −80 °C and thawed at a later time to ensure complete and homogeneous red blood cell hemolysis. Permeability changes in DSS models were performed (2 mg/kg and 6 mg/kg 800CW oral doses for 8 h and 14 h readouts, respectively). Permeability changes in CD40 models were performed (6 mg/kg 800CW) with blood sampling at 6 and 14 h for treatment and repeat-dosing studies, respectively. Blood was sampled on day 4 and day 7 in repeat dosing studies and day 7 for treatment studies. Blood samples were collected from DSS animals at predetermined timepoints over the course of two days to characterize the pharmacokinetics. Statistics were performed on GraphPad by one-way ANOVA with Tukey’s multiple comparisons test.

To explore 800CW permeability in non-human primates, data were generated using three cynomolgus macaques. Briefly, 800CW was orally administered at 1 mg/kg and 10 μL of collected whole blood at predetermined time points was used to characterize systemic 800CW signal over time. One primate had a history of colitis and had soft stool at the time of oral gavage dosing. The two other primates had normal bowel habits at the time of oral gavage dosing. Primate pharmacokinetic data were fit to a mechanistic 3-compartment model using MatLab (MathWorks, Natick, MA). Central and peripheral rate constants were previously calculated from intravenously dosed animals. With exchange rates between central and peripheral compartments constant, the net absorption rate, which includes small intestine and colon absorption, and colon elimination rate were fitted (Supplementary Fig. S11).

### Confocal laser endoscopy and ex vivo imaging

To assess the impact of disease and treatment on neutrophil activity, 4 nmol of NE680 was dosed intravenously on day 6 of DSS. Fourteen hours later, CLE was performed using a Cellvizio Dual Band system (Mauna Kea Technologies, Paris). Animals were lightly anesthetized with isoflurane and placed in a supine position under continuous oxygen/isoflurane. The colon was flushed with warm saline, followed by intra-rectally instilled acriflavine (100 μL of 0.05% wt/vol), and flushed again. Video acquisition was performed using the Ultra MiniO probe. A minimum of three videos covering the distal 2 cm of colon was captured per animal for analysis. Representative disease features (Fig. 3A) were assessed in each video and a score was assigned per animal depending on the quantity and distribution of key disease features using acriflavine staining and NE680 signal (Table 1). Statistics were performed on GraphPad by one-way ANOVA with Tukey’s multiple comparisons test. Immediately following CLE, animals were euthanized and colons were resected and imaged on a Li-Cor Pearl imager (Lincoln, NE). Mean fluorescence intensity (MFI) values for the regions of interest (ROIs) were measured to analyze the tissue activation of NE680, and transformed using min-max normalization.

### Histological assessment of epithelial damage

Colons were flushed with saline followed by fixation in 10% formalin. Following paraffin embedding, samples were sectioned at 5 μm and stained with H&E before being imaged using a Pannoramic 250 whole slide scanner at 20x magnification (3D Histech, Budapest, Hungary). Slides were measured for both distal and proximal epithelial erosion damage using CaseViewer (3D Histech, Budapest, Hungary). The distal 4 cm of colon were assessed microscopically for loss of superficial epithelium and the length of each erosion was summed for total erosion length. Inflammation and loss of crypts was quantified by measuring proximally from the rectal squamous junction the length of colon in which greater than 50% of normal crypt architecture was lost and replaced by inflammatory infiltrates and myofibroblasts. One-way ANOVA with Tukey’s multiple comparisons test was performed.

### Immunohistochemistry and image analysis

Immunohistochemistry was performed on de-paraffinized 5 μm sections using a Leica Bond RX Fully Automated Research Stainer (Leica Biosystems, Buffalo Grove, IL) at ambient temperatures unless otherwise indicated. Slides were incubated with Iba-1 Rabbit Polyclonal antibody followed by anti-rabbit poly-HRP-IgG and visualized with DAB.

Slides were also incubated with neutrophil elastase rabbit polyclonal antibody followed by poly-AP-anti-rabbit-IgG before being visualized with mixed red refine from Leica Red kit. Slides were scanned with the Pannoramic slide scanner to image DAB (Iba-1) and AP (NE). Image analysis was subsequently performed using Visiopharm software (Hoersholm, Denmark). For image analysis of Iba-1 and NE positive area in the mucosa, separate algorithms were used to calculate the area of the total mucosa (brown DAB for Iba-1 and red AP for NE). The ratio of the area occupied by the respective markers to the total mucosal area was then calculated as %Iba-1(+) and %NE(+) pixels, and transformed using min-max normalization. One-way ANOVA with Tukey’s multiple comparisons test was performed.

## Supplementary information

Supplementary information.

## Data Availability

The datasets generated during and/or analyzed during the current study are available from the corresponding author on reasonable request.
