# Supplementary material for: Near infrared readouts offer sensitive and rapid assessments of intestinal permeability and disease severity in inflammatory bowel disease models
Source: Sci Rep. 2020 Mar 13;10:4696. doi: 10.1038/s41598-020-61756-y (PMC7070059; doi:10.1038/s41598-020-61756-y)
Supplement: Supplementary file 1 — Supplementary information. [file 41598_2020_61756_MOESM1_ESM.pdf]

Supplementary information

Near infrared readouts offer sensitive and rapid assessments of intestinal permeability and disease severity in inflammatory bowel disease models

Liang Zhang[1], Craig D. Wallace[1], Jamie E. Erickson[1], Christine M. Nelson[1], Stephanie M. Gaudette[1], Calvin S. Pohl[2], Samuel D. Karsen[1], Gricelda H. Simler[2], Ruoqi Peng[1], Christopher A. Stedman[1], F. Stephen Laroux[1], Marc A. Wurbel[1], Rajesh V. Kamath[1], Bradford L. McRae[1], Annette J. Schwartz Sterman[1], and Soumya Mitra[1]*

[1]AbbVie Bioresearch Center, Worcester, MA 01605

[2]AbbVie Inc, North Chicago, IL 60064

Table of Contents

1. Probe molecular structure and synthesis and pharmacokinetics of 800CW-dextran

2. Animal body weight data

3. Immunohistochemistry stains and analysis

4. Model and fits for primate oral absorption

1. Probe molecular structure and synthesis and pharmacokinetics of 800CW-dextran

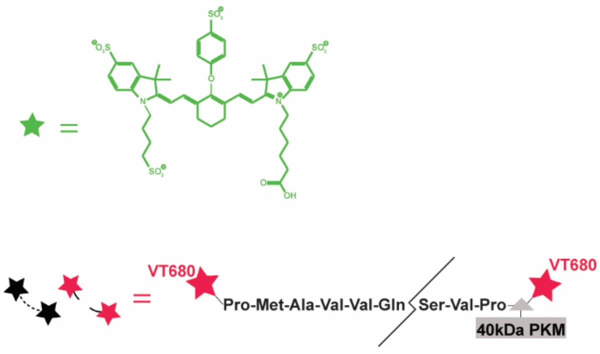

Figure S1. Molecular structure of IRDye 800CW and NE680 FAST.

3.5 kDa amino-dextran was obtained from FinaBio. To a solution of amino-dextran (200 nmol), 400 uL of 7.5% sodium bicarbonate was added. The mixture was stirred at room temperature for 1 min before addition of IRDye 800CW NHS ester (100 µmol). The reaction proceeded with gentle mixing at room temperature for 2 h before desalting using ZEBA desalting columns (7K MWCO) to remove unreacted dye. Due to the MWCO close to the dextran MW, there is reduced yield following purification.

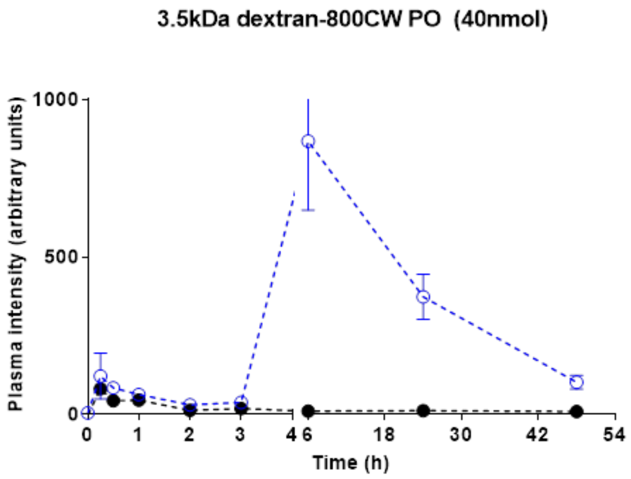

Figure S2. Pharmacokinetics of orally dosed 800CW-dextran in naive and DSS mice. Two distinctly separate phases are observed: one for stomach absorption and one for colon absorption (present in DSS treated animals only). 40 nmol dose referring to moles of fluorophore.

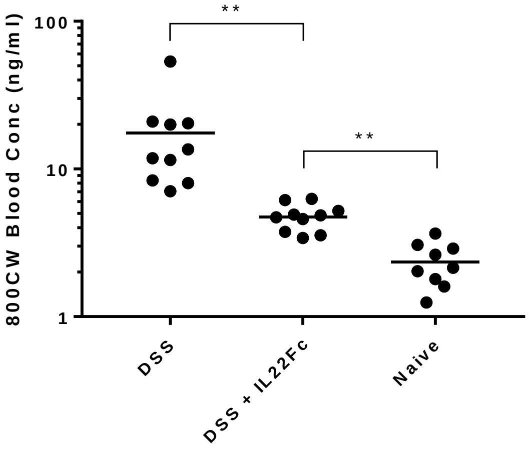

Figure S3. 800CW permeability detection in whole blood in mouse DSS. 120 nmol of 800CW carboxylate was orally gavaged 14 h before blood collection.

2. Animal body weight data

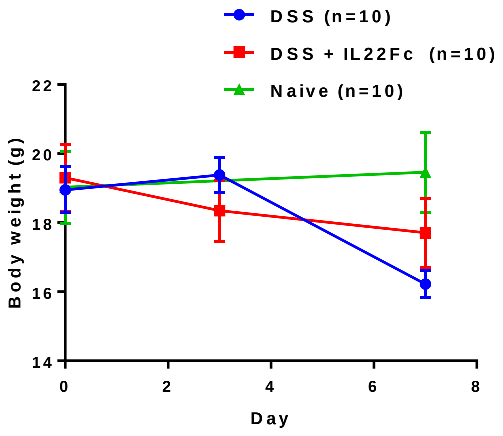

Figure S4. Animal weight data are recorded on day 0, 3, and 7. The data indicate body weight reduction in mice after 7 days of DSS treatment. This weight loss is reduced in the DSS + IL22Fc group. Animals without treatment increase in body weight over 7 days.

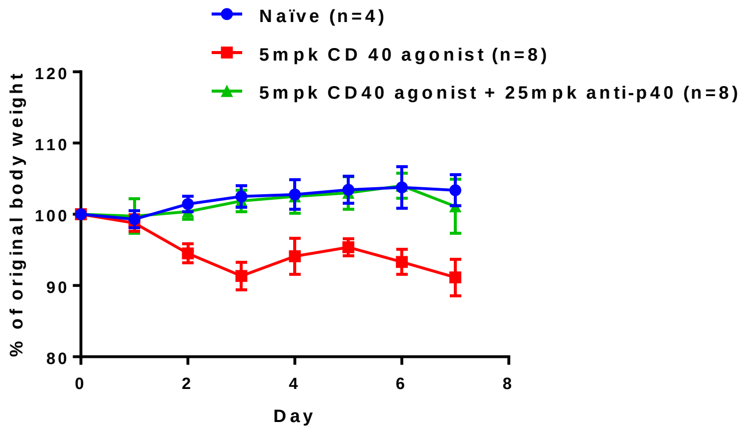

Figure S5. Body weight data for animal with anti-CD40, anti-CD40 + anti-p40, and naive treatments. The data show minimal changes in body for the anti-CD40 + anti-p40 group. Animals treated with 5 mg/kg anti-CD40 display statistically significant reductions in body weight up to one week following the initial anti-CD40 dose.

3. Immunohistochemistry stains and analysis

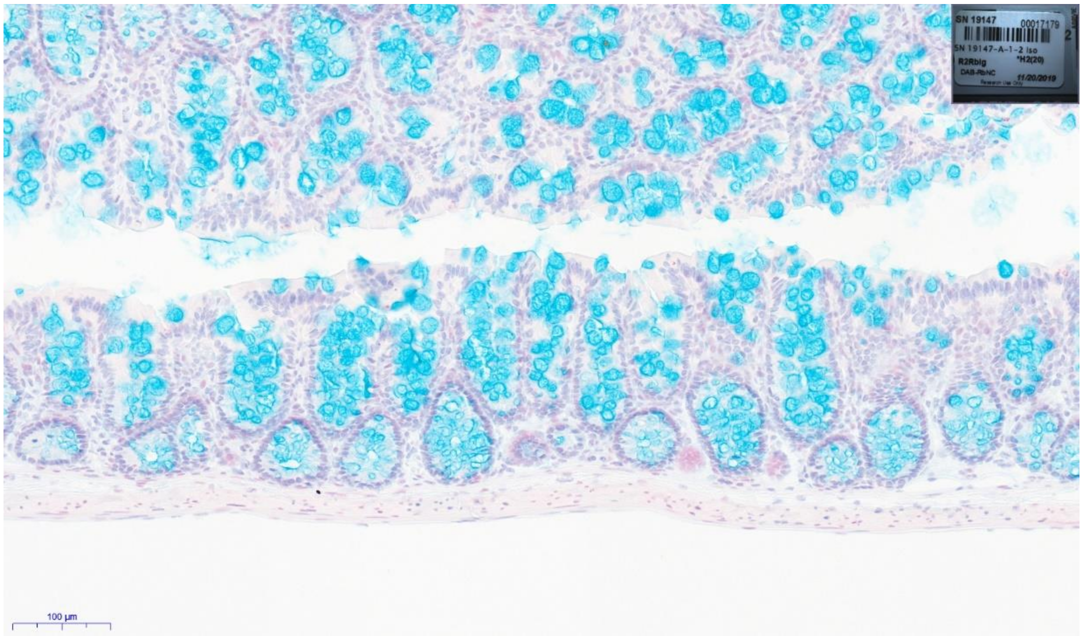

Figure S6. Mouse colon stained with Alcian blue for mucin. Briefly, the slide was washed in DI water, placed in 3% acetic acid for 3 min, and then stained with Alcian blue for 15 s. The slide was then rinsed with DI water 2x, before being counterstained with Nuclear Fast Red for 1 min.

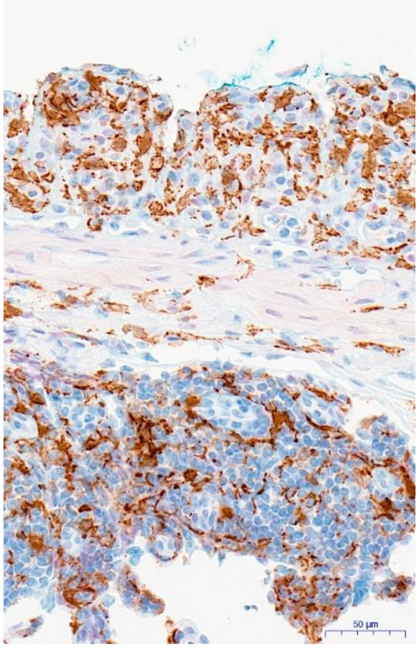

Figure S7. Single IHC stain for Iba-1 in mouse colon (DSS treatment, Iba-1 in brown; nuclear counterstain). Image analysis for Iba-1 was performed using two algorithms developed using the Visiopharm software. The first algorithm creates a mask for the mucosal tissue and the second algorithm identifies the Iba-1(+) pixels. Briefly, the tertiary lymphoid organs (TLO) that are

external to the muscularis mucosa and the rectum are removed from the total ROI, and the algorithms report fraction of Iba-1(+) pixel area in the mucosal tissue within the defined ROI.

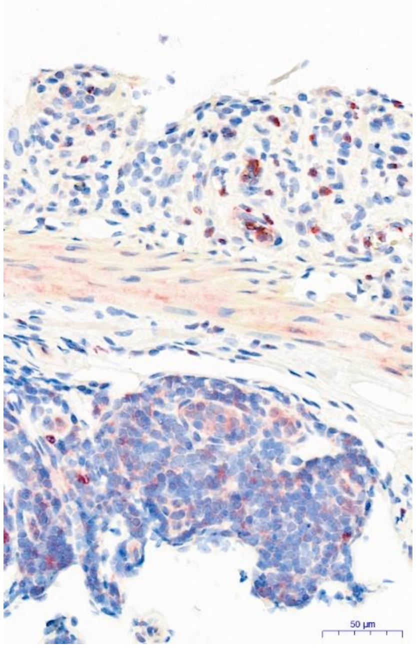

Figure S8. Single IHC stain in mouse colon for neutrophil elastase (DSS only). Image analysis algorithms identical to the one used for Iba-1 were applied to quantify the fraction of NE(+) pixels in the mucosa.

4. Model and fits for primate oral absorption

Feasibility of 800CW colon permeability was investigated in primates. We hypothesized minimal stomach and small intestine bioavailability due to molecular weight cutoff for oral absorption. However, experimentally, high oral bioavailability was observed in primates and a three compartment model was used to identify differences in net absorption rates[1,2] (Fig. S4).

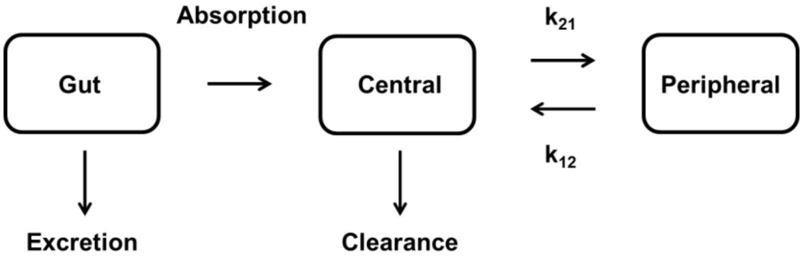

Figure S9. Three-compartment model with rate affecting oral absorption of IRDye 800CW

Intravenously dosed IRDye 800CW was dosed in mice and systemic pharmacokinetics was quantified using a LICOR Odyssey CLx. The IV data was fit to a two-compartment model similar to Fig. S9 (no digestive compartment) subject to

$$C(t) = C_0(Ae^{-k_\alpha t} + (1-A)e^{-k_\beta t})$$

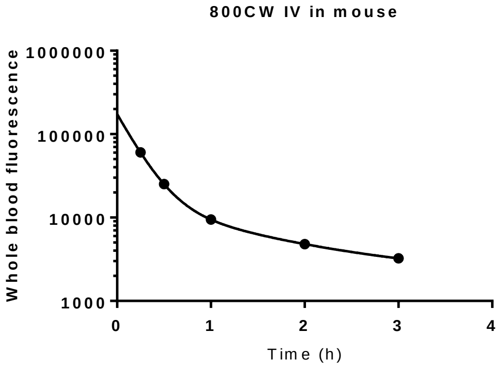

Figure S10. Biexponential decay following intravenous dose of 800CW carboxylate in mice.

Gut compartment balance (mass balance):

$$\frac{dX_{gut}}{dt} = -k_{abs}X_{gut} - k_{elcl}X_{gut}$$

With the initial condition at t=0, $X_{gut}=X_{gut,0}$=dose. The value of this initial condition is equivalent to the fluorescence intensity of the dose and is varied for each animal (dose is weight-dependent).

Central compartment balance:

$$\frac{dC_c}{dt} = k_{abs}C_{gut,0}e^{-(k_{abs}+k_{elcl})t} - k_{el}C_c - k_{12}C_c + k_{21}C_p$$

$$C_{gut,0} = \frac{X_{gut,0}}{V_{gut,0}}$$

Peripheral compartment balance:

$$\frac{dC_p}{dt} = k_{12}C_c - k_{21}C_p$$

Using central clearance rates and peripheral/central exchange rates from intravenously dosed 800CW, absorption (k_{abs}) and excretion rates (k_{elcl}) were fit simultaneously using MATLAB.

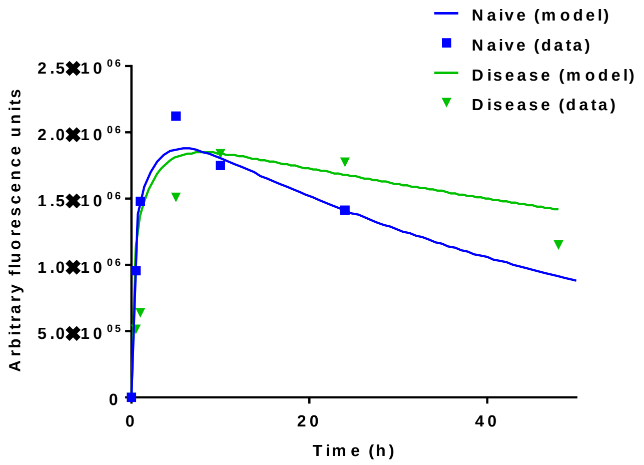

Figure S11. Experimental colon permeability (cynomolgus macaques) and model absorption data. The oral bioavailability of 800CW is significantly higher in primates compared to mice.

	k_{abs} (h^{-1})
Disease	.0035
Healthy	.0038

References

1. Zhang L, Navaratna T, Thurber GM. A Helix-Stabilizing Linker Improves Subcutaneous Bioavailability of a Helical Peptide Independent of Linker Lipophilicity. *Bioconjugate Chem*. 2016;27(7):1663-1672. doi:10.1021/acs.bioconjchem.6b00209

2. Li X, Li L, Zhou X, et al. Pharmacokinetic/pharmacodynamic studies on exenatide in diabetic rats. *Acta Pharmacol Sin*. 2012;33(11):1379-1386. doi:10.1038/aps.2012.33
